# Public Health Department

**Published:** 1900-02

**Authors:** I. J. Jones

**Affiliations:** Secretary Quarantine Department; Austin, Texas


### Public Health Department.

I. J. JONES, M. D., SECRETARY QUARANTINE DEPARTMENT.

[Each month, under this head, will be published all orders or instructions to county physicians promulgated from the office of the State Health Officer, together with abstracts of sanitary news and miscellany, by Dr. I. J. Jones, Secretary of the State Health Department.—ED.]

SOME STATISTICS, AND A MORAL.—State Health Officer Blunt has required the county health officers of the various counties to report the statistics of the various infectious diseases of their respective counties, for the period embraced between December 1, 1898, and August 1, 1899. While these statistics are not claimed to be altogether complete, they are doubtless approximately so, and so far as they extend are reliable.

Some very interesting facts are shown relating to smallpox and vaccination. Each county health officer was required to report the total number of cases of smallpox for his county; the total deaths; the number of cases occurring among those *recently* vaccinated. The total cases in those having scars of former vaccination, the number of deaths, among the two latter classes, etc.

Reports were received on this head from 157 counties of the State. Of these counties, 111 did not experience the disease, while 46 counties reported a total of 1,401 cases, which are tabulated as follows:

| | |
|---|---|
| Total number of cases among the unvaccinated | 1,324. |
| Total number of deaths among the unvaccinated | 230. |
| Mortality among the unvaccinated | 17.4 |
| Total number in those cases recently vaccinated | 57. |
| Total cases in those with scars of former vaccination | 20. |
| Total number in the vaccinated | 77. |
| Total deaths in the vaccinated | 5. |
| Mortality in the vaccinated | 6.5% |

Think of this! Notwithstanding the fact that a very large majority of our population are vaccinated, only one-eighteenth of the persons having smallpox had ever been vaccinated at all, and of this small number three-fourths had been recently vaccinated, i. e. (in most cases), after exposure to the disease. And this is not all; the percentage of mortality among the unvaccinated is almost three times as great as among the vaccinated. Could argument be stronger than these facts?

Look at it in another way. It has been carefully computed (not officially), that the counties, municipalities and State Quarantine Department have spent during these nine months no less than one hundred and fifty thousand dollars in the measures to suppress the disease, and the commercial loss was much greater.

Another fact developed in these reports as well as in the Marine Hospital reports is, that vaccination with glycerinized serum, which is perfectly aseptic, and when done with aseptic precautions is a *trivial matter,* a mere pin-scratch. Dr. E. E. Gwinn, county health officer of Cherokee county, vaccinated 800 convicts in the State penitentiary at Rusk, and of the entire number only two were disabled from work, and these only for one or two days. Why will not people be vaccinated, under such convincing circumstances? The answer is simple: Some few are deterred on account of having observed bad results follow the use of impure virus, arm to arm vaccination, or dirty methods. Quite a large number are so criminally ignorant as to neither know nor care anything about it; but the vast majority expect to have it done some time or other, but put it off from time to time.

We are all ready to exclaim: "Let's get the Legislature to pass a law making vaccination compulsory." Ay, there is the rub. Get them to do it if you can. Meanwhile, the Attorney-General of the State has ruled that the school trustees of any public school in the State have the right to make vaccination a prerequisite to attendance.

Now let us put our shoulders to the wheel and make life a burden to these same school trustees until they adopt such regulations. The negro and Mexican population of our State are principally responsible for spreading the disease, and represent a very large majority of the unvaccinated population. They are very prompt to avail themselves of public school privileges; let us see that they are vaccinated before they do it.

Smallpox is still reported from almost every State in the Union.

San Antonio is, so far as we know, the only city in the State that has a pathological laboratory connected with its health department. It is doing good service

Austin, Texas, January, 1900.

